# Antioxidative and Protective Effect of *Morchella esculenta* against Dextran Sulfate Sodium-Induced Alterations in Liver

**DOI:** 10.3390/foods12051115

**Published:** 2023-03-06

**Authors:** Shutong Chen, Min Wang, Suresh Veeraperumal, Bo Teng, Rui Li, Zhengming Qian, Jianping Chen, Saiyi Zhong, Kit-Leong Cheong

**Affiliations:** 1 Guangdong Provincial Key Laboratory of Aquatic Product Processing and Safety, Guangdong Province Engineering Laboratory for Marine Biological Products, Guangdong Provincial Engineering Technology Research Center of Seafood, Guangdong Provincial Science and Technology Innovation Center for Subtropical Fruit and Vegetable Processing, College of Food Science and Technology, Guangdong Ocean University, Zhanjiang 524088, China; 2 Department of Biology, College of Science, Shantou University, Shantou 515063, China; 3 Postgraduate College, Guangdong Ocean University, Zhanjiang 524088, China; 4 Dongguan HEC Cordyceps R&D Co., Ltd., Dongguan 523850, China

**Keywords:** *Morchella esculenta*, polysaccharide, antioxidant, ulcerative colitis, oxidative stress

## Abstract

*Morchella esculenta* is an edible mushroom with special flavor and high nutritional value for humans, primarily owing to its polysaccharide constituents. *M. esculenta* polysaccharides (MEPs) possess remarkable pharmaceutical properties, including antioxidant, anti-inflammatory, immunomodulatory, and anti-atherogenic activities. The aim of this study was to evaluate the in vitro and in vivo antioxidant potential of MEPs. In vitro activity was determined using free radical scavenging assays, whereas in vivo activity was evaluated through dextran sodium sulfate (DSS)-induced liver injury in mice with acute colitis. MEPs effectively scavenged 1,1-diphenyl-2-picrylhydrazyl and 2,2-azinobis-6-(3-ethylbenzothiazoline sulfonic acid) free radicals in a dose-dependent manner. Additionally, DSS-induced mice showed severe liver damage, cellular infiltration, tissue necrosis, and decreased antioxidant capacity. In contrast, intragastric administration of MEPs showed hepatoprotective effects against DSS-induced liver injury. MEPs remarkably elevated the expression levels of superoxide dismutase, glutathione peroxidase, and catalase. Additionally, it decreased malondialdehyde and myeloperoxidase levels in the liver. These results indicate that the protective effects of MEP against DSS-induced hepatic injury could rely on its ability to reduce oxidative stress, suppress inflammatory responses, and improve antioxidant enzyme activity in the liver. Therefore, MEPs could be explored as potential natural antioxidant agents in medicine or as functional foods to prevent liver injury.

## 1. Introduction

Oxidation is essential for the production of energy to fuel biological processes and for physiological functions in living organisms. However, excessive production of oxygen-derived free radicals or a reduction in antioxidant levels leads to oxidative stress [1]. Oxidative stress is a deleterious process that can considerably damage cell structure, including lipids, proteins, DNA, and RNA, leading to many diseases. Antioxidants play a vital role in protecting the body from excessive oxidative damage linked to cancer, cardiovascular diseases, chronic inflammation, liver diseases, and neurodegenerative diseases [2]. Oxidative stress plays an important role in ulcerative colitis (UC) initiation and progression. UC is an inflammatory bowel disease and chronic and nonspecific inflammatory disorder that primarily involves the mucosa and submucosa of the colon. In patients with UC that is characterized by highly potent reactive oxygen species (ROS) and decreased antioxidant levels in the colonic mucosa, oxidative stress could ultimately contribute to chronic tissue damage [3]. The drugs used to treat UC have many undesirable gastrointestinal and liver side effects, including diarrhea, abdominal pain, fatigue, and hepatic fibrosis [4]. Therefore, there is an increasing interest in using natural sources to extract effective and safe antioxidants that can protect the human body from free radicals and treat UC without side effects.

In recent years, the development of nutraceutical and pharmaceutical activities has increasingly focused on the active components derived from natural resources, owing to their higher therapeutic effects and acceptable toxicological profile. *Morchella esculenta* belongs to the *Helvellaceae* family of fungi and is a commercially available edible mushroom with a honeycomb shape and delicious taste. *M. esculenta* is a globally distributed species found in temperate regions of Europe, Asia, North America, and Australia. It commonly grows during the spring season, between March and May, in forested areas with moist soil and abundant organic matter [5]. Since ancient times, it has been widely used for culinary and medicinal purposes. Traditional Chinese medicine attributes morels to the lung, liver, and kidney meridians [6]. Today, modern pharmaceutical research has demonstrated that *M. esculenta* mushrooms exhibit a wide range of biological activities, such as antioxidant, antitumor, anti-inflammatory, immunoregulatory, nephroprotective, anti-hyperlipidemic, and anti-atherosclerosis activities [7]. As morels have various health advantages, the market requirement for *M. esculenta* has increased accordingly. Similarly, new cultivation technologies have been developed to meet the increasing demand for this mushroom [8].

The fruiting bodies of *M. esculenta* possess various active constituents, including tocopherols, carotenoids, organic acids, phenolic compounds, and polysaccharides. Polysaccharides are formed by long chains of monosaccharides linked by glycosidic bonds. The health benefits of polysaccharides have been appreciated for a long time. These molecules are the primary bioactive constituents of *M. esculenta* and have been highlighted for their remarkable pharmaceutical activities, including antioxidant [9], anti-inflammatory [10], immunomodulatory [11], and anti-atherogenic activities [12]. Many parameters of research on *M. esculenta* polysaccharide MEPs have included extraction processes, chemical–physical properties, different collected regions, and fermentation conditions [9,13]. These parameters all contribute to the theoretical basis of the structure–function relationship of MEPs. Furthermore, the antioxidant effects of polysaccharides have attracted considerable attention in pharmaceutical and function food research for their defense against oxidative stress. Clinical evidence has shown the important role of oxidative stress in several tumors and cardiovascular diseases [14,15]. The hot water extraction of MEP has been found to possess free radical scavenging activity and has demonstrated significant acetylcholinesterase and butyryl cholinesterase inhibition activities [9]. Additionally, a 959 kDa molecular weight of MEP, isolated from an aqueous extract of *M. esculenta* fruiting bodies, has been shown to alleviate liver injury in chronic alcohol-induced mice [16]. Enzyme-assisted MEP from the fruiting bodies has been reported to significantly improve liver function in high-fat high-cholesterol emulsion-induced hyperlipidemia mice [17]. However, the effectiveness of MEP in mitigating the effects of Dextran sulfate sodium (DSS)-induced colitis-induced oxidative stress and liver injury has not been thoroughly examined.

Therefore, the aim of this study was to evaluate the radical-scavenging activity of MEPs in vitro. In addition, in vivo antioxidant activity was evaluated by measuring superoxide dismutase (SOD), glutathione peroxidase (GSH-Px), catalase (CAT), myeloperoxidase (MPO) activity, and malondialdehyde (MDA) levels in the context of secondary liver caused by DSS-induced colitis. This study sheds new light on the potential therapeutic benefits of MEPs for liver health and provides fundamental research on the potential use of MEPs as healthy food ingredients and dietary supplements.

## 2. Materials and Methods

### 2.1. Materials

*M. esculenta* was purchased from a local market (Guangdong, China). All standards were purchased from Sigma-Aldrich (St. Louis, MO, USA); dextran sulphate sodium (DSS) was purchased from Aladdin (Beijing, China). The SOD, GSH-Px, and MDA commercially available kits were purchased from Solarbio Science & Technology Company (Beijing, China). The CAT and MPO commercially available kits were purchased from Elabscience Biotechnology Company (Wuhan, China). All the other chemicals and solvents used in this experiment were of analytical grade.

### 2.2. Preparation of the MEPs

According to the property of polysaccharides, we used water-extraction and alcohol-precipitation method to obtain MEPs [18]. The dried *M. esculenta* was first decolorized by adding anhydrous ethanol; then, the powder was dried again naturally and was subsequently sifted through an 80-mesh sieve. The obtained powder was stored in a dryer before using. During extraction, we suitably put the powder into a beaker and added distilled water at a ratio of 1:40 (*w*/*v*), followed by stirring and heating in a 90 °C water bath for 2 h. After cooling, the solution was divided into tubes and centrifuged at 4000 rpm for 10 min. The collected supernatant was concentrated and 95% ethanol was added at a volume ratio of 1:3 for the precipitate of polysaccharides. The precipitate was collected via centrifugation and redissolved in distilled water. The polysaccharide solution was then subjected to a DEAE-52 cellulose column eluted by distilled water and dialyzed with a molecular weight cut-off of 3500 Da dialysis bag for 24 h. After dialysis, the polysaccharide solution was freeze-dried, and finally, the MEPs were obtained.

### 2.3. Molecular Weight Determination and Monosaccharide Composition Analysis

The molecular weight of MEPs was determined by high-performance gel permeation chromatography (HPGPC) following the method of Li et al. [19] with a slight modification. Briefly, the MEPs samples were dissolved in distilled water to obtain a solution of 5 mg/mL; then, the solution was filtered through a 0.22 μm Millipore membrane and restored in a clean 1.8 mL sample vial before detection. The instrument was equipped with a refractive index detector (RI-10A) using a BRT105-104-102 tandem gel column (8 × 300 mm i.d.) (Borui Saccharide, Biotech. Co. Ltd, Yangzhou, China). Next, 20 μL solution was injected, with a mobile phase of 0.05 mol/L NaCl at a speed of 0.6 mL/min, and the temperature of the column was 40 °C. Standard dextrans of known molecular weight (5000, 11,600, 23,800, 48,600, 80,900, 148,000, 273,000, 409,800, 667,800) were used to make the standard curve, and the molecular weight of MEPs was calculated.

Monosaccharide composition of MEPs was analyzed by using an ion chromatograph (IC) (Thermo Fisher Scientifific, Waltham, MA, USA) according to the method of Hui et al. [20] with a minor modification. Specially, a sample of 5 mg MEPs was hydrolyzed in 3 mol/L trifluoroacetic acid (TFA) at 120 °C for 3 h; then, the hydrolysate was transferred into a tube and dried by N2 gas flow. After drying, 5 mL of distilled water was added into the tube, and the solution was vortexed until even. The obtained homogeneous solution was diluted 20 times with distilled water and then centrifuged at 12,000 rpm for 5 min. The supernatant was taken to be analyzed by IC with a Dionex CarbopacTMPA20 column (3 × 150 mm). The condition was set as follows: mobile phase was composed of H2O (A), 15 mmol/L NaOH (B), and 15 mmol/L NaOH and 100 mmol/L NaAC (C); flow rate was 0.3 mL/min; sample size was 5 μL; column temperature was 30 °C; sample was detected through an electrochemical detector.

### 2.4. In Vitro Radical Scavenging Activities

The analysis of 1,1-diphenyl-2-picrylhydrazyl (DPPH) radical scavenging activity followed a previous study with slight modification [21]. In brief, aliquots of polysaccharide solution (0.1 mL) at varying concentrations (1.0–3.0 mg/mL) were mixed with a solution of 50 mmol/L DPPH in ethanol (0.1 mL). Subsequently, the mixture was reacted in the dark for 0.5 h, and the absorbance was determined by an enzyme microplate reader at 517 nm. Ascorbic acid was used as the positive control in this assay. The calculating formula is as follows:

$$\;\mathrm{Scavenging}\;\mathrm{activity}\left(\%\right)=\left(1-\frac{A_{1}-A_{2}}{A_{0}}\right)\times 100\%$$

where A_0_ is the absorbance values of blank, A_1_ is the absorbance values of polysaccharides; A_2_ is the absorbance values of negative control (ethanol instead of DPPH solution).

The analysis of 2,2-azinobis-6-(3-ethylbenzothiazoline sulfonic acid) (ABTS)radical scavenging activity was performed according to a previous report [22]. In brief, an aliquot of 7 mmol/L of ABTS solution (5 mL) and 2.45 mmol/L of potassium persulfate (88 μL) was mixed together at room temperature for 16 h. Then, 2 mL of ABTS radical solution was mixed with polysaccharide sample solutions at varying concentrations (1.0–3.0 mg/mL). After reacting for 10 min in the dark, the absorbance was also measured by an enzyme microplate reader at 734 nm. Ascorbic acid was used as the positive control. The calculating formula is as follows:

$$\;\mathrm{Scavenging}\;\mathrm{activity}\left(\%\right)=\left(1-\frac{A_{1}-A_{2}}{A_{0}}\right)\times 100\%$$

where A_0_ is the absorbance values of blank, A_1_ is the absorbance values of polysaccharides, A_2_ is the absorbance values of the negative control (distilled water instead of ABTS solution).

### 2.5. Animal Experiment and Sample Collection

Animal experiments were conducted according to the regulation from Guangdong Ocean University Laboratory Animal Centre (GDOU-LAE-2022-028). Six-week-old female C57BL/6 mice (weighted 35–38 g) were purchased from Guangdong Sijia Jingda Biotechnology Limited Company (Guangzhou, China). After a one-week accommodation (temperature at 23 °C ± 2 °C, humidity at 50 ± 10%, 12 h light/dark cycle), the mice were randomly divided into 4 groups, including control group (control, *n* = 5), model group (DSS, *n* = 5), mice treated with MEP group (DSS + MEP, *n* = 5), and mice treated with salazosulfapyridine (SASP)group (DSS + SASP, *n* = 5). The control group mice drank water ad libitum and were regularly treated with food of commercial-grade animal chow for 3 weeks, while the other three groups were handled differently. Except for the control group, the other three groups of mice were induced with 3% DSS (*w*/*v*) aqueous solution to build a colitis model for the first week. Then, for the final two weeks of the experiment, the DSS group was treated the same as the control group, and the DSS+MEP group and DSS+SASP group were intragastrical administered with MEP and SASP, both at a dose of 0.1 mg/g bw. During the experiment, body weight, the condition of diarrhea and stool bleeding were recorded. In the end, all mice were sacrificed, and the following samples were immediately collected for further experiments. The organs of each group were collected and weighted, including the liver, spleen and kidney. The organ index was calculated by using the formula:

$$\mathrm{Organ}\;\mathrm{Index}\;(\%)=\frac{\mathrm{the}\;\mathrm{weight}\;\mathrm{of}\;\mathrm{organ}}{\mathrm{the}\;\mathrm{weight}\;\mathrm{of}\;\mathrm{mouse}\;}\times 100\%$$

Especially, part of liver was washed by ice-cold PBS solution to remove the fat and blood and was then processed for biochemical analyses, and the rest of it was fixed for histological analyses.

### 2.6. Histological Evaluation

A histological evaluation of the liver of the four groups was performed with a light microscope. Briefly, the collected liver samples were fixed in 4% paraformaldehyde solution for H&E staining, and we used a light microscope to observe the changes of hepatocytes in each group; then, photos were taken (400×).

### 2.7. Determination of the SOD, GSH-Px and CAT Activities

The collected liver samples and extracting solution were mixed at a ratio of 1:9 (*w*/*v*) and then homogenized by an ultrasonic processor for 1 min. The homogenate was centrifuged at 10,000 g for 10 min at 4 °C to obtain the supernatant. The supernatant was placed on ice for the determination of antioxidant status. The protein content of the liver sample was measured by bicinchoninic acid (BCA) protein assay. The activity of SOD, GSH-Px, and CAT was determined according to the instructions of the commercially available kit and was expressed as enzyme activity (U) per mg protein.

### 2.8. MPO Activity and MDA Content

The above liver homogenate supernatant was used to estimate the MPO activity and MDA content by using commercially available kits. The MPO activity was expressed as enzyme activity (U) per gram of tissue, and the content of MDA, which was produced by lipid peroxidation, was expressed as nmol MDA per milligram of protein.

### 2.9. Statistical Analysis

All the data were analyzed by mean ± SD (standard deviation). Significant differences between the experimental groups were determined by the multiple comparison of one-way ANOVA (GraphPad prism 9.3 software), and differences were considered statistically significant at *p* < 0.05.

## 3. Results and Discussion

### 3.1. Molecular Weight and Monosaccharide Composition of MEPs

**Figure 1:**
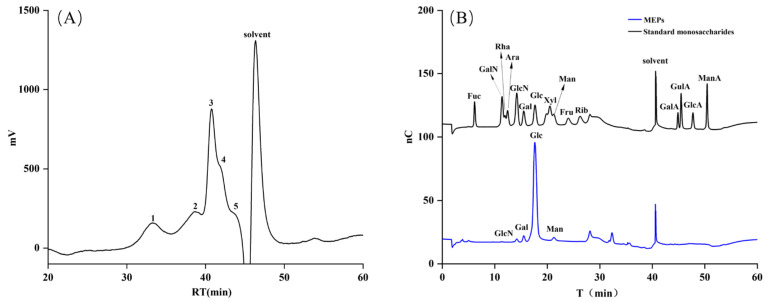
Molecular weight and monosaccharide composition analysis results of MEPs, HPGPC profile (**A**) and ion chromatography (IC) profile (**B**). (Fuc: fucose; GalN: galactose hydrochloride; Rha: rhamnose; Ara: arabinose; GlcN: glucosamine hydrochloride; Gal: galactose; Glc: glucose; Xyl: xylose; Man: mannose; Fru: fructose; Rib: ribose; GalA: galactose acid; GulA: guluronic acid; GlcA: glucuronic acid; ManA: mannuronic acid.)

Molecular weight distribution and monosaccharide composition are both crucial variables related to the bioactivity of polysaccharides; thus, it is vital for us to clarify the structure–function relationship of the macromolecule [23,24]. The molecular weight distribution and property of MEPs are shown in Figure 1A and Table 1. There are five portions of MEPs, and the molecular weight averages (Mw), molecular weight number averages (Mn), and the molecular weight of each highest peak (Mp) of the maximum component were 19.714 kDa (38.873%), 14.330 kDa (38.873%), and 16.930 kDa (38.873%), respectively, which demonstrate that MEPs have a lower relative molecular weight and a wide molecular weight distribution.

The quality of polysaccharides can be measured by analyzing the monosaccharide contents. The monosaccharide composition of MEPs are shown in Figure 1B and Table 1. In terms of peak time, the corresponding monosaccharides from front to back are: GalN, Gal, Glc and Man, which are compared with standard monosaccharides. Thus, the IC results show that MEPs are composed of glucosamine hydrochloride, galactose, glucose and mannose in the molar ratio of 0.009:0.053:0.900:0.037, and the main monosaccharide component is glucose.

### 3.2. Effects of MEP Antioxidant Properties In Vitro

**Figure 2:**
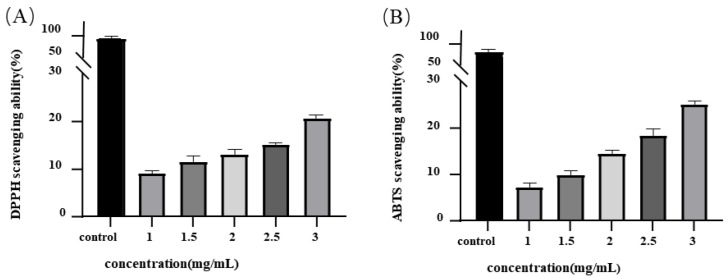
Scavenging capacities of MEP on DPPH radical (**A**) and ABTS radical (**B**).

Free radicals and related species are generated by several endogenous systems under various physicochemical conditions and pathological states in the human body. When produced in excess, free radicals can stimulate oxidative stress, which can considerably alter cell membranes and other cellular structures, causing various disease states [25]. Dietary antioxidants scavenge excess free radicals to protect cells against toxic effects and to prevent the development of disease. In this study, we used DPPH and ABTS scavenging assays to evaluate the in vitro antioxidant activities of MEPs. The ability of MEP to scavenge DPPH radicals is shown in Figure 2A. The DPPH radical scavenging activity of MEPs increased from 9.23% to 20.73% when the concentration ranged from 1.0 to 3.0 mg/mL. Although MEP scavenging activity correlated with increasing concentration, it was significantly lower than that of ascorbic acid. The DPPH scavenging activity of the crude extract of *M. esculenta* at a concentration of 20 μg/mL was found to be 39.7%. This is higher than our observed results, which may be attributed to the presence of 47.01 μg/mg of polyphenols in the extract [26]. Polyphenols are known to be strong antioxidant compounds and could have contributed to the higher scavenging activity observed in the *M. esculenta* crude extract. The DPPH radical scavenging activity reported in our study is lower than that reported by Cai et al. [27], wherein the IC_50_ value for scavenging DPPH radicals of polysaccharides extracted from cultured *M. esculenta* was 1.090 mg/mL. This may be due to the collection of *M. esculenta* from different regions and culture conditions. MEP has been demonstrated to have the ability to interact with free radicals and to act as a scavenger to prevent oxidative damage. This property is attributed to the presence of hydroxyl groups within MEP, which are capable of donating electrons to free radicals and stabilizing them. Typically, the effect of polysaccharides on DPPH radical scavenging activity is based on their hydrogen-donating capacity.

The ABTS assay is another effective method for determining the total antioxidant power of components. This method defines the component potential for H-atom donation and chain-breaking. As shown in Figure 2B, MEPs scavenged ABTS radicals in a concentration-dependent manner (7.37–25.33% at 1.0–3.0 mg/mL). Li et al. [28] purified MEPs from unfermented soybean curd residue. Their study demonstrated that these polysaccharides can scavenge hydroxyl radicals at concentrations between 0.156 and 10 mg/mL, whereas the same result could not be achieved with ascorbic acid of the same concentration. The mechanism underlying the ABTS radical scavenging capacity of MEPs is similar to that of the DPPH radical scavenging. Both are mainly involved in the reaction and rapidity of H-atom transfer. The hydroxyl groups in polysaccharides (normally at the C-2 and C-6 positions) participate in H-atom transfer reactions toward the ABTS radical [29,30]. Here, it was hypothesized that MEPs donate electrons and hydrogen atoms to unstable ABTS^+^ cation radicals to yield a stable ABTS^-^ polysaccharide form. The study proposed a mechanism underlying the antioxidant capacity of MEPs through in vitro experiments and provided novel insights into the potential therapeutic applications of MEPs for liver diseases. To further support the evidence of improvement, an in vivo study was also conducted.

### 3.3. Effects of MEPs on Organ Indices in Mice

**Figure 3:**
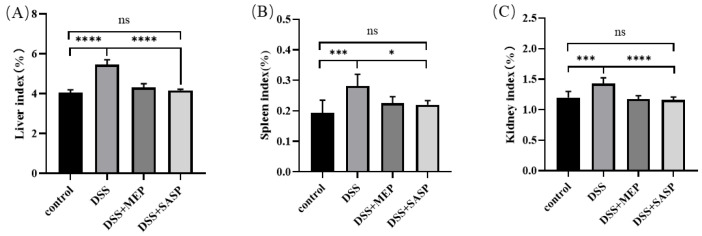
The effect of MEP on the liver index (**A**), spleen index (**B**), and kidney index (**C**). The values are expressed as mean ± SD (*n* = 5). * (*p* ≤ 0.05), *** (*p* < 0.001), and **** (*p* < 0.0001) are in comparison with each group, ns means no significance.

Liver injury is one of the most common complications in patients with ulcerative colitis (UC). Therefore, it is necessary to identify functional food-based drugs or nutraceuticals that can effectively improve liver damage in enteritis models and reduce the risk of hepatic diseases [31]. In this study, a mouse colitis model was developed by administering 3% DSS to induce adverse liver injury. As shown in Figure 3A, the liver index in the DSS-induced mouse group was significantly higher than that in the control group. Hepatic diseases are often associated with an increase in liver index, which is a valuable index for assessing liver injury. MEP treatment restored the liver index, which decreased from 5.45% to 4.30%.

The spleen is among the important lymphatic organs containing various immune cells, such as B cells, T cells, NK cells, and macrophages. Its index reflects inflammation levels and typical indicators of UC. Here, the spleen index was significantly higher in the DSS-induced group than in the control group (Figure 3B). However, treatment of DSS-induced mice with MEPs considerably decreased the spleen index. The increase in spleen weight is due to immune activation or infection, leading to hyperactivity. Similarly, the kidney index can be used to measure the severity of inflammation. In this study, the kidney index result was the same as that of the spleen index (Figure 3C). That is, the kidney index also showed a significant increase in the DSS-induced group compared to the other three groups. Therefore, changes in spleen and kidney indices reflect innate immune function. Our results indicate that MEPs could potentially alleviate secondary liver injury in mice with DSS-induced UC.

### 3.4. MEP Treatment Attenuated DSS-Induced Hepatitis

**Figure 4:**
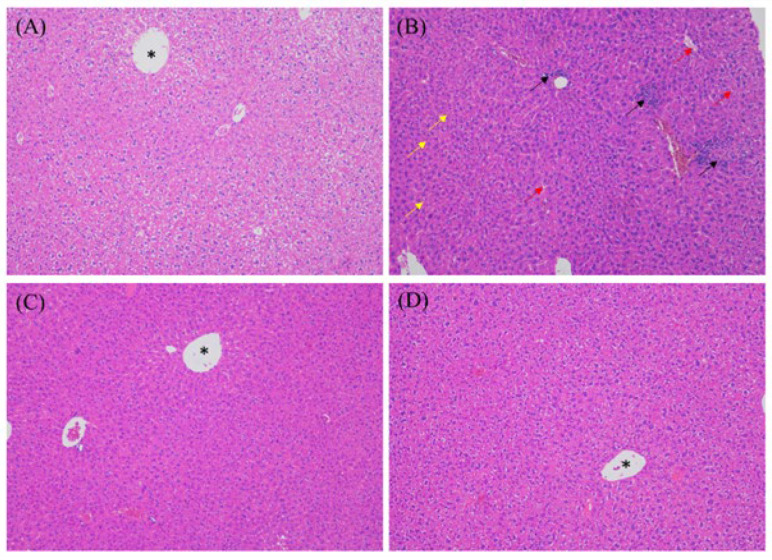
Representative photomicrographs of liver tissues were obtained from normal control group (**A**), the DSS-induced mice group (**B**), the DSS-induced mice treated with MEP (**C**), and the DSS-induced mice treated with SASP (**D**). The red arrows represent the hepatocytes with clear vacuoles, the yellow arrows stand for the pyknotic nuclei, and the black arrows represent the noticeable accumulation of inflammatory cells in the vicinity of the central vein (*) (H&E, ×400).

H&E staining was used to examine liver histology (Figure 4). The stained tissue slices showed that the livers of DSS-induced mice had an indistinct or irregular arrangement of cell borders. Additionally, these results revealed vacuolar degeneration of hepatocytes, some of which appeared to have a distinct appearance and displayed inflammatory cell infiltration, pyknotic nuclei, and hepatocyte necrosis (Figure 4B). MEP-treated mice showed clear cell membranes and distinct cell edges. Hepatocyte necrosis and inflammatory cell infiltration were attenuated after MEP treatment (Figure 4C). These results demonstrate that MEPs have a protective effect against liver damage in DSS-induced mouse models. Owing to the essential metabolic function of the liver, the consequent liver damage may cause severe health concerns. In the DSS-induced mouse models, liver damage was caused by cellular damage, oxidative stress, inflammation, and cholestasis [32]. Although the mechanisms underlying tissue regeneration are not completely established, MEPs substantially alleviate cellular injury and inflammatory response in the liver. Meng et al. [33] demonstrated that the hepatoprotective effects of *M. esculenta* against alcohol-induced acute liver injury in C57BL/6 mice may be related to the modulation of anti-inflammatory and antioxidant signaling pathways, which are related to Nrf-2 and NF-κB signaling.

### 3.5. Anti-Oxidative Effects Contribute to MEP-Mediated Hepatoprotection

**Figure 5:**
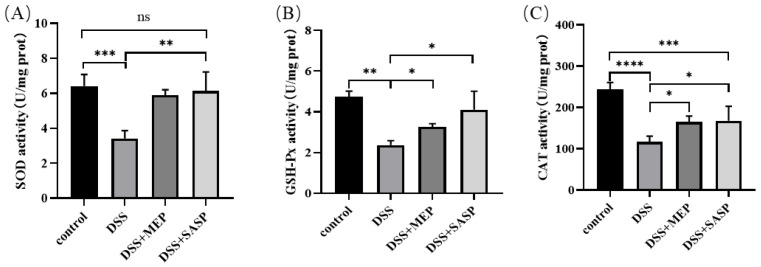
Effect of MEP on oxidative stress in DSS-induced mice. SOD activity (**A**), GSH-Px activity (**B**) and CAT activity (**C**). * (*p* ≤ 0.05), ** (*p* < 0.01), *** (*p* < 0.001), and **** (*p* < 0.0001) are in comparison with each group, ns means no significance.

Oxidative stress plays a crucial role in the development and progression of colitis-induced liver diseases [34]. Excessive ROS formation can induce oxidative stress that oxidizes proteins, DNA, RNA, and carbohydrates, leading to hepatocyte damage. The liver is equipped with an antioxidant protection system that protects against free radicals through enzymatic and non-enzymatic mechanisms. Among the enzymes with strong antioxidant properties is SOD. Here, liver SOD activity was analyzed to assess the mice antioxidative properties. The SOD activity in the DSS-induced group was considerably lower than that in the control group. However, MEP administration substantially increased the SOD activity in this group. Similarly, SASP administration significantly increased SOD activity (Figure 5A). GSH-Px is another oxidation indicator that protects against oxidative damage. It does so by promptly removing lipid peroxide and hydrogen peroxide using glutathione as a reductant. This enzyme also reduces the generation of hydroxyl free radicals [35]. As shown in Figure 5B, the control group had the highest GSH-Px levels, whereas the DSS-induced group had the opposite result. However, MEP considerably increased GSH-Px levels. In a similar study, SOD and GSH-Px expression was downregulated in DSS-induced colitis. Furthermore, antioxidant agents considerably relieved colitis and colitis-associated secondary liver damage [36].

CAT is the main enzyme involved in the decomposition of hydrogen peroxide (H_2_O_2_) and reactive nitrogen species. This enzyme plays an important role in cellular and tissue protection against oxidative stress [37]. Similar to SOD, CAT activity was suppressed in DSS-induced mice. However, it was enhanced by MEP treatment (Figure 5C). This indicates that the livers of mice with UC were damaged by oxidation, whereas MEPs reversed this trend. SOD, CAT, and GSH-Px are regarded as the first line of the antioxidant defense system against free radicals produced in the liver during oxidative stress. These enzymes can operate individually or act cooperatively under different sites in the metabolic pathway of free radicals. Our results demonstrate that MEPs can improve antioxidant function by promoting antioxidant enzyme activity, which can provide a first line of defense against free radicals produced in the liver during oxidative stress.

### 3.6. MEP Suppressed DSS-Induced Oxidative Stress

**Figure 6:**
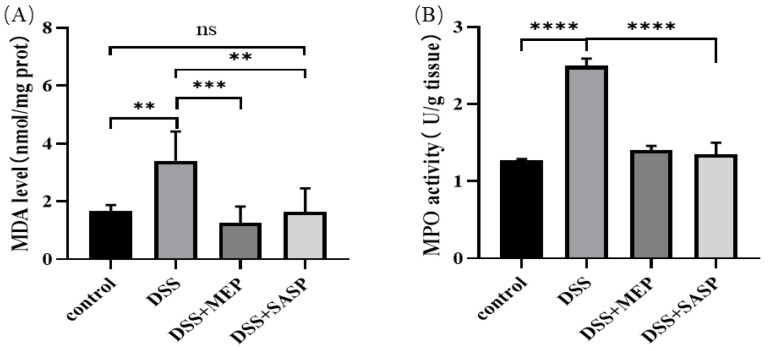
MEP suppressed DSS-induced oxidative stress. MDA level (**A**) and MPO activity (**B**). ** (*p*< 0.01), *** (*p* < 0.001), and **** (*p* < 0.0001) are in comparison with each group, ns means no significance.

MDA is generated in vivo through the peroxidation and decomposition of polyunsaturated fatty acids. The compound is a common indicator of increased ROS production and may reflect oxidative damage to liver tissues [38]. The MDA level in the DSS-induced group was higher than that in the control group. However, MEPs substantially reduced MDA levels in DSS-induced mice (Figure 6A). MPO is a heme-containing enzyme that is abundantly stored in azurophilic granules of human inflammatory cells. This enzyme contains peroxidase, which generates a range of ROS by catalyzing halide anions in the presence of H_2_O_2_ to form a corresponding hypohalous acid. Excessive production of hypohalous acids, especially during chronic inflammation, may result in oxidative damage and the formation of halogenated products [39]. In this study, MPO activity in the liver homogenate was evaluated. As shown in Figure 6B, MPO activity decreased to 1.40 ± 0.06 U/g tissue after MEP treatment at the 100 mg/kg body weight dose, which was lower than that in the DSS-induced group (2.50 ± 0.09 U/g tissue). Similarly, MEPs extracted from fruiting bodies and degraded using snailase decreased MPO activity (0.90 ± 0.05 U/g) at the 400 mg/kg body weight dose when compared with high-fat high-cholesterol emulsion-induced hyperlipidemia mice [17]. The in vivo results obtained here provide new insights into the potential therapeutic benefits of MEPs for liver health.

## 4. Conclusions

In this study, the antioxidant activity of MEPs was investigated in vitro and in vivo. DPPH and ABTS assays demonstrated that MEP possesses good scavenging capabilities. These results suggest that MEPs have potent antioxidant properties in vitro. Intragastric administration of MEPs attenuates DSS-induced hepatic pathological damage. Here, MEPs increased SOD, CAT, and GSH-Px activities and decreased MDA and MPO levels in the liver of DSS-induced mice. These results indicate that MEPs can substantially increase antioxidant properties by enhancing antioxidant enzyme activities. Moreover, these polysaccharides have a potential role in improving liver tissue damage and the antioxidant defense system induced by DSS. Therefore, our results suggest that MEPs could be regarded as a potential natural antioxidant agent in the functional foods and pharmaceutical industries. In addition, they can be used as a novel hepatoprotective agent for the prevention of liver injury. However, further investigation is required to define how MEPs modulate specific gut microbiota and microbiota-derived functional metabolites correlated with liver protection.

## Figures and Tables

**Table 1:** Physicochemical properties of MEPs.

Indices	RT (min)	Relative Molecular Weight (kDa)			Relative Percentage of Peak Area (%)
		Mw	Mn	Mp	
Molecular weight	(1) 33.298	504.275	289.188	354.204	15.739
	(2) 38.700	48.229	32.837	39.183	19.984
	(3) 40.759	19.714	14.330	16.930	38.874
	(4) 41.697	13.115	9.822	11.551	18.435
	(5) 43.322	6.473	5.105	5.957	6.967
	Monosaccharide				Molar ratio
Monosaccharide composition	GlcN				0.009
	Gal				0.053
	Glc				0.900
	Man				0.037

## Data Availability

The data are available from the corresponding author.

## References

[1] Radi R. (2018). Oxygen radicals, nitric oxide, and peroxynitrite: Redox pathways in molecular medicine. Proc. Natl Acad. Sci. USA.

[2] Fridovich I. (1999). Fundamental aspects of reactive oxygen species, or what’s the matter with oxygen?. Ann. N. Y. Acad. Sci..

[3] Pereira C., Grácio D., Teixeira J.P., Magro F. (2015). Oxidative stress and DNA damage: Implications in inflammatory bowel disease. Inflamm. Bowel Dis..

[4] Rogler G. (2010). Gastrointestinal and liver adverse effects of drugs used for treating IBD. Best Pract. Res. Clin. Gastroenterol. GA..

[5] Du X.H., Zhao Q., Yang Z.L. (2015). A review on research advances, issues, and perspectives of morels. Mycology.

[6] Sunil C., Xu B. (2022). Mycochemical profile and health-promoting effects of morel mushroom *Morchella esculenta* (L.)—A review. Food Res. Int..

[7] Li I.C., Chiang L.H., Wu S.Y., Shih Y.C., Chen C.C. (2022). Nutrition profile and animal-tested safety of *Morchella esculenta* mycelia produced by fermentation in bioreactors. Foods.

[8] Liu Q., Ma H., Zhang Y., Dong C. (2018). Artificial cultivation of true morels: Current state, issues and perspectives. Crit. Rev. Biotechnol..

[9] Badshah S.L., Riaz A., Muhammad A., Tel Çayan G., Çayan F., Emin Duru M., Ahmad N., Emwas A.-H., Jaremko M. (2021). Isolation, characterization, and medicinal potential of polysaccharides of *Morchella esculenta*. Molecules.

[10] Li W., Cai Z.N., Mehmood S., Liang L.L., Liu Y., Zhang H.Y., Chen Y., Lu Y.M. (2019). Anti-inflammatory effects of *Morchella esculenta* polysaccharide and its derivatives in fine particulate matter-treated NR8383 cells. Int. J. Biol. Macromol..

[11] Zhang N.N., Ma H., Zhang Z.F., Zhang W.N., Chen L., Pan W.J., Wu Q.X., Lu Y.M., Chen Y. (2022). Characterization and immunomodulatory effect of an alkali-extracted galactomannan from *Morchella esculenta*. Carbohydr. Polym..

[12] Wang D., Yin Z., Ma L., Han L., Chen Y., Pan W., Gong K., Gao Y., Yang X., Chen Y. (2021). Polysaccharide MCP extracted from *Morchella esculenta* reduces atherosclerosis in LDLR-deficient mice. Food Funct..

[13] Li J., Wu H., Liu Y., Nan J., Park H.J., Chen Y., Yang L. (2021). The chemical structure and immunomodulatory activity of an exopolysaccharide produced by *Morchella esculenta* under submerged fermentation. Food Funct..

[14] Yao W., Qiu H.M., Cheong K.L., Zhong S. (2022). Advances in anti-cancer effects and underlying mechanisms of marine algae polysaccharides. Int. J. Biol. Macromol..

[15] Cheong K.L., Yu B., Chen J., Zhong S. (2022). A comprehensive review of the cardioprotective effect of marine algae polysaccharide on the gut microbiota. Foods.

[16] Teng S., Zhang Y., Jin X., Zhu Y., Li L., Huang X., Wang D., Lin Z. (2023). Structure and hepatoprotective activity of Usp10/NF-kappaB/Nrf2 pathway-related *Morchella esculenta* polysaccharide. Carbohydr. Polym..

[17] Dong Y., Qi Y., Liu M., Song X., Zhang C., Jiao X., Wang W., Zhang J., Jia L. (2018). Antioxidant, anti-hyperlipidemia and hepatic protection of enzyme-assisted *Morehella esculenta* polysaccharide. Int. J. Biol. Macromol..

[18] Wu D.T., An L.Y., Liu W., Hu Y.C., Wang S.P., Zou L. (2022). In vitro fecal fermentation properties of polysaccharides from *Tremella fuciformis* and related modulation effects on gut microbiota. Food Res. Int..

[19] Li R., Zhou Q.L., Chen S.T., Tai M.R., Cai H.Y., Ding R., Liu X.F., Chen J.P., Luo L.X., Zhong S.Y. (2022). Chemical characterization and immunomodulatory activity of fucoidan from *Sargassum hemiphyllum*. Mar. Drugs.

[20] Hui Y., Jun-Li H., Chuang W. (2019). Anti-oxidation and anti-aging activity of polysaccharide from *Malus micromalus Makino* fruit wine. Int. J. Biol. Macromol..

[21] Cheong K.L., Li J.K., Zhong S. (2022). Preparation and structure characterization of high-value *Laminaria digitata* oligosaccharides. Front. Nutr..

[22] Khan B.M., Qiu H.M., Xu S.Y., Liu Y., Cheong K.L. (2020). Physicochemical characterization and antioxidant activity of sulphated polysaccharides derived from *Porphyra haitanensis*. Int. J. Biol. Macromol..

[23] Yao W., Gong Y., Li L., Hu X., You L. (2022). The effects of dietary fibers from rice bran and wheat bran on gut microbiota: An overview. Food Chem. X.

[24] Wang M., Veeraperumal S., Zhong S., Cheong K.L. (2023). Fucoidan-derived functional oligosaccharides: Recent developments, preparation, and potential applications. Foods.

[25] Zheng L.X., Liu Y., Tang S., Zhang W., Cheong K.L. (2023). Preparation methods, biological activities, and potential applications of marine algae oligosaccharides: A review. Food Sci. Hum. Well..

[26] Wagay J.A., Nayik G.A., Wani S.A., Mir R.A., Ahmad M.A., Rahman Q.I., Vyas D. (2019). Phenolic profiling and antioxidant capacity of *Morchella esculenta* L. by chemical and electrochemical methods at multiwall carbon nanotube paste electrode. J. Food Meas. Charact..

[27] Cai Z.N., Li W., Mehmood S., Pan W.J., Wang Y., Meng F.J., Wang X.F., Lu Y.M., Chen Y. (2018). Structural characterization, in vitro and in vivo antioxidant activities of a heteropolysaccharide from the fruiting bodies of *Morchella esculenta*. Carbohydr. Polym..

[28] Li S., Sang Y., Zhu D., Yang Y., Lei Z., Zhang Z. (2013). Optimization of fermentation conditions for crude polysaccharides by *Morchella esculenta* using soybean curd residue. Ind. Crops Prod..

[29] Yuan Y., Xu X., Jing C., Zou P., Zhang C., Li Y. (2018). Microwave assisted hydrothermal extraction of polysaccharides from *Ulva prolifera*: Functional properties and bioactivities. Carbohydr. Polym..

[30] Wang M., Cheong K.L. (2023). Preparation, structural characterisation, and bioactivities of fructans: A review. Molecules.

[31] Restellini S., Chazouillères O., Frossard J.L. (2017). Hepatic manifestations of inflammatory bowel diseases. Liver Int..

[32] Li L., Wang Y., Zhao L., Ye G., Shi F., Li Y., Zou Y., Song X., Zhao X., Yin Z. (2022). Sanhuang xiexin decoction ameliorates secondary liver injury in DSS-induced colitis involve regulating inflammation and bile acid metabolism. J. Ethnopharmacol..

[33] Meng B., Zhang Y., Wang Z., Ding Q., Song J., Wang D. (2019). Hepatoprotective effects of *Morchella esculenta* against alcohol-induced acute liver injury in the C57BL/6 mouse related to Nrf-2 and NF-κB signaling. Oxid. Med. Cell. Longev..

[34] Liang J., Chen S., Hu Y., Yang Y., Yuan J., Wu Y., Li S., Lin J., He L., Hou S. (2018). Protective roles and mechanisms of *Dendrobium officinal* polysaccharides on secondary liver injury in acute colitis. Int. J. Biol. Macromol..

[35] Brigelius-Flohé R., Maiorino M. (2013). Glutathione peroxidases. BBA—Gen. Subjects.

[36] Dziąbowska-Grabias K., Sztanke M., Zając P., Celejewski M., Kurek K., Szkutnicki S., Korga P., Bulikowski W., Sztanke K. (2021). Antioxidant therapy in inflammatory bowel diseases. Antioxidants.

[37] Yoo J.H., Erzurum S.C., Hay J.G., Lemarchand P., Crystal R.G. (1994). Vulnerability of the human airway epithelium to hyperoxia: Constitutive expression of the catalase gene in human bronchial epithelial cells despite oxidant stress. J. Clin. Investig..

[38] Del Rio D., Stewart A.J., Pellegrini N. (2005). A review of recent studies on malondialdehyde as toxic molecule and biological marker of oxidative stress. Nutr. Metab. Cardiovasc. Dis..

[39] Kalyanaraman B. (2013). Teaching the basics of redox biology to medical and graduate students: Oxidants, antioxidants and disease mechanisms. Redox Biol..

